# Osteogenic Effects of Ion Released from Biodegradable Metallic Magnesium and Calcium Coating

**DOI:** 10.1002/adbi.202500401

**Published:** 2025-11-12

**Authors:** Risa Miyake, Masaya Shimabukuro, Masahiko Terauchi, Eriko Marukawa, Masakazu Kawashita

**Affiliations:** ^1^ Department of Regenerative and Reconstructive Dental Medicine Graduate School of Medical and Dental Sciences Institute of Science Tokyo 1‐5‐45 Yushima Bunkyo‐ku Tokyo 113‐8549 Japan; ^2^ Department of Inorganic Biomaterials Laboratory for Biomaterials and Bioengineering Institute of Integrated Research Institute of Science Tokyo 2‐3‐10, Kanda‐Surugadai Chiyoda‐ku Tokyo 101‐0062 Japan

**Keywords:** biodegradable metals, dental implants, ion release, osteoblasts, osteogenesis, pH elevation

## Abstract

This study investigates the effects of ion release and pH elevation from a biodegradable metallic magnesium (Mg) –30 calcium (Ca) coating on osteogenesis using osteoblast‐like cells. The coating, formed on titanium (Ti) via magnetron sputtering, has previously been shown to enhance osteogenesis by promoting calcite formation on the Ti surface upon degradation in vitro study. However, the individual and combined roles of released Mg^2+^, Ca^2+^, and pH elevation remain unclear. To clarify these effects, culture media supplemented with Mg^2+^ and Ca^2+^ salts are prepared. Mg^2+^ at 4–5 mm promotes early alkaline phosphatase (ALP) activity compared to the 0.9 mm control, without affecting proliferation but suppressing mineralization. Ca^2+^ at 2.3–3 mm enhances ALP activity without affecting proliferation or mineralization compared to the 1.3–2.2 mm control. When both ions coexist, proliferation, ALP activity, and mineralization are enhanced compared to Mg^2+^ alone, suggesting a synergistic effect. Furthermore, the elevated pH resulting from the Mg–30Ca extract more effectively promotes proliferation, accelerates the peak of ALP activity, and supports mineralization than ions co‐supplementation. These findings indicate that Mg–30Ca coatings enhance osteogenesis through both ion release and pH elevation, providing new insight into the osteogenic potential of biodegradable metallic coatings.

## Graphical abstract

This study explores how magnesium–calcium coatings on titanium promote osteogenesis by releasing ions and elevating pH during degradation. Using osteoblast‐like cells, the research demonstrates that both ion release and pH changes contribute to cell proliferation, differentiation, and mineralization, offering new insights into the design of bioactive, biodegradable implant coatings for enhanced bone formation.

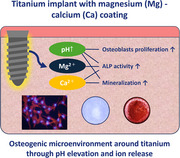

## 1 Introduction

Biodegradable metallic materials have gained increasing attention in recent years as advanced biomaterials for bone regeneration. Unlike inert materials, these metals gradually dissolve in the body and release ions that can actively participate in bone healing.^[^ ^1^, ^2^ ^]^ Among them, magnesium (Mg)‐based alloys have emerged as particularly promising candidates due to their suitable mechanical properties, biocompatibility, and osteogenic activities.^[^ ^3^, ^4^, ^5^ ^]^ Mg is an essential trace element involved in various cellular processes, and its ion presence has been shown to stimulate bone cell proliferation, differentiation, and mineralization.^[^ ^6^, ^7^, ^8^ ^]^ In addition, calcium (Ca), another essential element for osteogenic cell responses, is often considered in combination with Mg to further enhance osteogenic potential.^[^ ^9^, ^10^ ^]^ Despite these biological advantages, biodegradable metallic materials are generally not suitable as bulk components for dental implants, as their gradual degradation leads to loss of structural integrity over time. In addition, their mechanical strength is typically insufficient to withstand the high occlusal forces generated during mastication, limiting their application in load‐bearing regions.

Titanium (Ti) and its alloys remain the gold standard for dental implants due to their excellent biocompatibility. First, they possess sufficient mechanical properties to withstand the high occlusal forces generated during mastication.^[^ ^11^ ^]^ Second, they exhibit high corrosion resistance and biological inertness, primarily due to the formation of a stable titanium dioxide passive layer.^[^ ^12^ ^]^ This bioinert surface contributes to minimizing adverse reactions in surrounding tissues and allows for direct structural and functional integration with bone, a process known as osseointegration.^[^ ^13^ ^]^ However, because the untreated Ti surface does not actively promote cellular responses, osseointegration typically requires a prolonged healing period until the implant becomes firmly anchored in bone and can support a prosthetic superstructure to restore masticatory function. Therefore, surface modification of Ti is essential for enhancing its osteoconductivity and promoting stable, long‐term integration with the surrounding bone tissue.^[^ ^14^, ^15^ ^]^

To address this issue, bioactive surface coatings are required to enhance the osteoconductivity of Ti implants while preserving their inherent mechanical strength and biocompatibility. This strategy is expected to improve the biological response at the bone–implant interface and accelerate early healing, potentially reducing the overall treatment period.^[^ ^16^ ^]^ To date, we have developed a biodegradable Mg–30Ca‐based coating on Ti surfaces using magnetron sputtering with an Mg–30Ca alloy target. The resulting layer is composed of metallic Mg and Ca and exhibits good adhesive strength to the Ti surface. Moreover, it rapidly dissolved under physiological conditions, subsequently forming a calcium carbonate (calcite) layer on the surface.^[^ ^17^ ^]^ This calcite formation significantly promoted osteoblast proliferation and mineralization. Thus, we demonstrated that it is possible to promote osteogenesis by functionalizing Ti surfaces through the dissolution of biodegradable Mg–30Ca coatings formed on the Ti substrates. This finding suggests that, when applied to dental implants, Mg–30Ca coatings may accelerate osseointegration, allowing earlier fixation to the jawbone and potentially shortening the healing period before prosthetic loading. On the other hand, since metallic Mg and Ca in the layer dissolve rapidly, local conditions near the coating, such as ion concentration and pH, are also expected to change. Nevertheless, the effects of ion release from the coating and the consequent pH shifts induced by rapid dissolution have yet to be thoroughly investigated. This change in the local microenvironment may influence cell behavior, independently or synergistically with the released ions. While Mg^2+^ and Ca^2+^ are individually known to support osteogenesis,^[^ ^6^, ^7^, ^8^, ^9^, ^10^ ^]^ the biological consequences of their simultaneous release—and whether any synergistic or antagonistic effects exist—remain poorly understood. In the present study, we sought to simulate the post‐degradation environment of the coating by using extraction solutions containing the ions released from the Mg–30Ca coating. This approach allowed us to independently assess the effects of Mg^2+^, Ca^2+^, and pH changes on the proliferation, differentiation, and mineralization of osteoblast‐like cells, providing fundamental insight into the mechanism of biodegradable Mg–Ca coatings for dental implants. The novelty of this study lies in simulating the alkaline microenvironment formed by Mg–30Ca dissolution and providing new insights into the application of Mg‐based biodegradable materials to dental implants—a field where clinical use has been very limited. Therefore, this study aimed to elucidate the interactive effects of Mg, Ca, and pH elevation on osteogenic responses, thereby clarifying how the microenvironment generated by Mg–30Ca dissolution contributes to early bone formation on Ti surfaces.

## 2 Materials and Methods

### 2.1 Coating Preparation

As described in our previous study, commercially pure Ti discs (grade 2, 10 mm × 1.5 mm) were polished, cleaned, and coated with an Mg–30Ca alloy by magnetron sputtering in an Ar atmosphere.^[^ ^17^, ^18^ ^]^ Mg–30Ca coated Ti disks were provided by Maruemu Works (Osaka, Japan).

### 2.2 Characterization of the Mg–30Ca Coating

The thickness of the Mg–30Ca coating was determined by stylus profilometry. The surface roughness (Ra) of Mg–30Ca coating was measured using a laser scanning confocal microscope (VK‐9710, Keyence, Osaka, Japan) at × 50 magnification, corresponding to a scan area of ≈287.1 × 215.6 mm^2^ (n = 3). Scanning electron microscopy with energy‐dispersive X‐ray spectrometry (SEM/EDS; JSM‐IT200LA, JEOL, Tokyo, Japan) was used to observe the surface elemental distribution.

To investigate the effect of dissolution on pH, Mg–30Ca‐coated Ti disks were immersed in 1 mL of an alpha modification of Eagle's minimum essential medium (MEMα; Fujifilm Wako Pure Chemical, Osaka, Japan) supplemented with 10% fetal bovine serum (FBS; HyCloneTM, Cytiva, Logan, UT, USA), 100 U mL^−1^ penicillin, 100 µg mL^−1^ streptomycin, and 0.25 µg mL^−1^ amphotericin B (FUJIFILM Wako). The subsequent experiments were performed using the supplemented medium. They were incubated in an incubator at 37 °C under a 5% CO_2_ atmosphere. After 24 h of incubation, the pH of the extraction medium was measured using the pH meter (LAQUAtwin; HORIBA, Kyoto, Japan). The medium was then replaced with a fresh 1 mL of medium, and the pH was measured again after an additional 24 h. This procedure was repeated every 24 h up to 96 h to monitor temporal changes in the pH of the extraction medium during coating dissolution. Untreated Ti disks were used as controls, and all experiments were performed in triplicate (n = 3). In addition, for the ion‐supplemented groups listed in **Table** **1** (Mg, Ca, and MgCa), 500 µL of medium was incubated in an incubator at 37 °C under a 5% CO_2_ atmosphere for 24 h, and the pH was measured using a pH meter (HORIBA). The medium without any ion supplementation was used as the control, and all experiments were performed in quintuplicate (n = 5).

**Table 1:** Mg^2+^ and Ca^2+^ concentration and pH of each medium used in the cell viability test.

Samples	Mg^2+^ concentration [mm]	Ca^2+^ concentration [mm]	pH
Control	0.9	2.2	8
Mg	5	2.2	8
Ca	0.9	3	8
MgCa	5	3	8
MC30	5	3	9.5

### 2.3 Cytotoxicity Test

As described in our previous study, MC3T3‐E1 cells (Riken BioResource Center) were cultured in the medium.^[^ ^19^ ^]^ These cells were maintained at 37 °C in a humidified atmosphere containing 5% CO_2_. MgCl_2_∙6H_2_O (FUJIFILM Wako) was added to the cell culture medium to adjust the Mg^2+^ ions concentration to 5, 10, 50, or 100 mm. Non‐adjusted medium was used as a control (0.9 mm). For cytotoxicity assessment, ≈10000 cells mL^−1^ of MC3T3‐E1 cells were cultured in the medium containing 0.9, 5, 10, 50, and 100 mm Mg^2+^ using 48 wells. After 1, 3, and 5 days of incubation, the number of viable cells in each sample (n = 5) was determined using water‐soluble tetrazolium salt (Cell Counting Kit‐8; CCK‐8; DOJINDO, Kumamoto, Japan). Cells were incubated with 10% (v/v) CCK‐8 reagent in the culture medium for 60 min at 37 °C, and absorbance was measured at 450 nm using a microplate reader. The obtained absorbance values were converted to cell density using a calibration curve prepared in advance. The subsequent cell proliferation assay was performed using the same protocol.

### 2.4 Cell Viability Test

The Mg–30Ca‐coated Ti disk was immersed in the medium at 37 °C for 24 h in a humidified atmosphere containing 5% CO_2_. Thus, Mg^2+^ and Ca^2+^ of the coating were extracted into the medium. The extraction was quantified using inductively coupled plasma‐atomic emission spectrometry (ICP‐AES; ICPE‐9800; Shimadzu Corp., Kyoto, Japan). The actual Mg^2+^ and Ca^2+^ concentrations of the cell culture medium and the extraction of Mg–30Ca coating using this medium are shown in Table S1 (Supporting Information). Based on the measured ion concentrations of this extract, the Mg–30Ca extract group (MC30) was defined as having an Mg^2+^ concentration of ≈5 mm and a Ca^2+^ concentration of 3 mm. In this study, as shown in Table 1, samples were prepared by adding Mg or Ca salts to the culture medium so that the Mg^2+^ and Ca^2+^ concentrations were equivalent to those in the MC30. The amounts of salts were calculated and added accordingly to achieve these final concentrations. The Mg, Ca, and MgCa groups were prepared by supplementing the medium with each Mg^2+^ and Ca^2+^ concentration using MgCl_2_·6H_2_O and CaCl_2_·2H_2_O (Fujifilm Wako). The MC30 group did not contain any added Mg or Ca salts; all ions were derived solely from the Mg–30Ca coating extract. The control group consisted of the original cell culture medium. The pH of each medium was analyzed by pH meter (HORIBA).

For cell viability assessment, ≈10000 cells mL^−1^ of MC3T3‐E1 cells were cultured in each medium using 48 wells. After 1 and 5 days of incubation, the number of viable cells in each sample (n = 5) was determined CCK‐8 (DOJINDO). The adhered cells were rinsed with PBS and fixed in 10% formalin for 20 min on ice after 1 and 5 days of incubation. Furthermore, the adhering cells were permeated and blocked by immersing the samples in 0.5% Triton X‐100 (Sigma–Aldrich) for 15 min, rinsed again with PBS. The actin and nuclei of the attached cells were stained with 100 nm of Acti‐stain 555 phalloidin (Cytoskeleton, Inc., Denver, CO, USA) for 30 min at room temperature, and 10 µg mL^−1^ of Hoechst 33342 (DOJINDO) for 30 s at room temperature. Fluorescent images were captured using an X71 microscope and a DP70 charge‐coupled device camera (Olympus, Tokyo, Japan). Excitation/emission filters of 350/461 nm for Hoechst 33 342 and 535 ± 20/585 ± 20 nm for phalloidin were used. Images were obtained using a 10× objective lens.

To evaluate the independent effect of pH elevation on cell proliferation, MC3T3‐E1 cells were cultured in the medium with different ionic compositions and pH conditions. The ion‐supplemented groups were prepared as shown in Table 1. In addition, media with the same ion concentrations were further adjusted to pH9.5 using 1 m NaOH to reproduce the alkaline condition observed in the Mg–30Ca extract. Unmodified medium was used as the control. Approximately 10000 cells mL^−1^ of MC3T3‐E1 cells were cultured in each medium at 37 °C in a humidified atmosphere containing 5% CO_2_. After 1 and 5 days of incubation, the number of viable cells in each sample (n = 5) was determined using CCK‐8 (DOJINDO).

### 2.5 Osteogenic Evaluations

To induce osteogenic differentiation, the cell culture medium supplemented with 10 mmol L^−1^ β‐glycerophosphate (Fujifilm Wako), 50 µg mL^−1^ L‐ascorbic acid (FUJIFILM Wako), and 150 nmol L^−1^ dexamethasone (FUJIFILM Wako) was used (differentiation‐inducing medium). Mg–30Ca‐coated Ti disk was extracted in the differentiation‐inducing medium. The extract was subsequently sterilized by filtration. The filtration was performed to remove precipitates formed in the medium. The actual Mg^2+^ and Ca^2+^ concentrations of the differentiation‐inducing medium and the extraction of Mg–30Ca coating using this medium are shown in Table S2 (Supporting Information). Based on these data, the amounts of salts were calculated and added to achieve final concentrations of ≈4 mm Mg^2+^ and 2.3 mm Ca^2+^. The Ca^2+^ concentration of the medium decreased because of filtering, so the concentrations of Mg^2+^ and Ca^2+^ were modified as described in **Table** **2**. The control group consisted of the differentiation‐inducing medium as a control. The Mg, Ca, and MgCa groups were prepared by supplementing the differentiation medium with each Mg^2+^ and Ca^2+^ concentrations using MgCl_2_·6H_2_O and CaCl_2_·2H_2_O. The pH of each medium was analyzed by a pH meter (HORIBA).

**Table 2:** Mg^2+^ and Ca^2+^ concentrations and pH of each medium used in the osteogenic evaluations.

Samples	Mg^2+^ concentration [mm]	Ca^2+^ concentration [mm]	pH
Control	0.9	1.3	8
Mg	4	1.3	8
Ca	0.9	2.3	8
MgCa	4	2.3	8
MC30	4	2.3	8.7 (ALP) 9.3 (ARS)

Alkaline phosphatase (ALP) activity was determined to evaluate the osteogenic differentiation of MC3T3‐E1 cells in each sample after 4 and 7 days of incubation. Instead of the media used in Alizarin Red S (ARS) evaluation, pH‐adjusted MC30 was used. MC3T3‐E1 cells were incubated in these media using 48wells. The medium was changed after every 4 days. MC3T3‐E1 cells on the samples after 4 and 7 days of incubation were stained using Alkaline Phosphatase Staining Kit (Cosmo Bio, Tokyo, Japan) based on 5‐bromo‐4‐chloro‐3‐indolyl‐phosphate/nitro blue tetrazolium (BCIP/NBT). The cells were rinsed with PBS, fixed in 4% formalin for 20 min, and stained with ALP at 37 °C for 10 min. The optical microscope images of cells on the samples were obtained, and the percentage area of ALP staining on the samples was measured using a BZ‐X700 microscope and digital analysis software (BZ‐X; Keyence, Osaka, Japan). The ALP‐stained area was calculated by ImageJ software (Wayne Rasband, National Institutes of Health, Bethesda, MD, USA) (n = 3‐4).^[^ ^20^ ^]^

To verify the appropriateness of these time points, additional ALP staining was performed for the control group at culture days 5, 10, and 15 (n = 3). In addition, to evaluate the independent effect of pH on ALP activity, MC3T3‐E1 cells were cultured for 5 days in control medium and in control medium adjusted to pH 8.7 using 1 m NaOH. After incubation, the cells were stained for ALP activity using the same method described above (n = 3).

For assessment of calcified matrix formation, MC3T3‐E1 cells were incubated in these media using 48wells. The medium was changed every 4 days. After 21 days of incubation, the mineralization of MC3T3‐E1 cells was evaluated by observing the color change of the calcified deposits after ARS staining (Sigma‐Aldrich, St. Louis, MO, USA). The medium was removed, and the attached cells were rinsed with PBS. The cells were fixed in 10% formalin for 20 min on ice. The samples were then rinsed with ultrapure water. Each sample was stained with 1% ARS solution (adjusted to pH 6.4 with ammonium hydroxide) at 25 °C for 5 min.^[^ ^21^ ^]^ The cells were repeatedly rinsed with ultrapure water after removing the ARS solution. When the samples were completely dry, their surface was observed using an S9D microscope (Leica, Wetzlar, Germany) equipped with a Digital Sight 1000 camera (Nikon, Tokyo, Japan). The ARS‐stained area was calculated by ImageJ software (National Institutes of Health) (n = 3–4 independent samples per group). In addition, to evaluate the independent effect of pH on ARS staining, MC3T3‐E1 cells were cultured for 21 days in control medium adjusted to pH 9.3 using 1 m NaOH. After incubation, the cells were stained for calcified area using the same method described above (n = 3).

### 2.6 Statistical Analysis

Appropriate statistical analyses are described in the figure legends, along with the number of independent experiments performed. In summary, all statistical analyses were performed using Kaleida Graph software version 5.0.6 (Synergy Software, Eden Prairie, MN, USA). Possible outliers were evaluated using the Smirnov–Grubbs rejection test before statistical analysis. Data are presented as mean ± SD. *P*‐values are calculated using one‐way ANOVA followed by Tukey's post hoc test. * represents *p *< 0.05, ** represents *p *< 0.01 compared to the control group at each time point. Horizontal bars indicate significant differences among other groups, where applicable.

## 3 Results

### 3.1 Characterization

**Figure 1:**
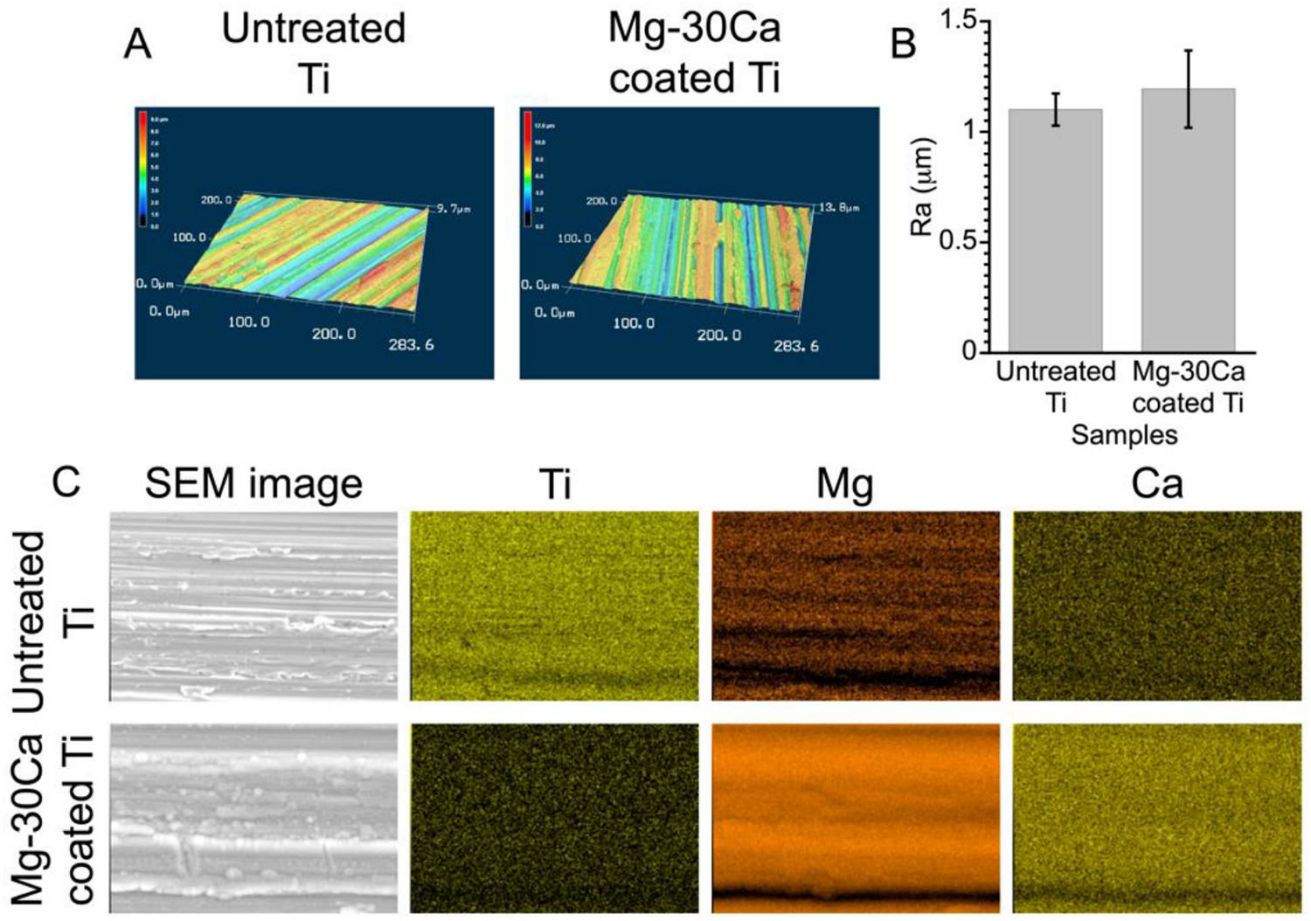
Surface characterization of the untreated Ti and Mg–30Ca‐coated Ti. 3D laser microscopy images A), the surface roughness B), and scanning electron microscopy (SEM) images and mapping images of Ti, Mg, and Ca C) of untreated Ti and Mg–30Ca coated Ti. Data presented as mean ± SD, n = 3, *P*‐values are calculated using one‐way ANOVA followed by Tukey's post hoc test. Significance was defined as *p *< 0.05.

The thickness of the MC30 coating was 9.8 µm, and the surface roughness (Ra) value of Mg–30Ca‐coated Ti was 1.2 µm. In comparison, the Ra of untreated Ti was 1.1 µm, and no significant difference was observed between the two groups (**Figure** **1**A,B). These results indicate that the Mg–30Ca coating was deposited along the underlying surface morphology of the substrate. SEM images and Ti, Mg, and Ca elemental mapping images of Mg–30Ca coated Ti disk and untreated Ti disk are shown in Figure 1C. The EDS mapping of Mg–30Ca coated Ti disk revealed that Mg and Ca elements were uniformly distributed along the polished surface of the Ti substrate. The pH of the extract from the Mg–30Ca coated Ti reached its maximum value (≈8.3) after 24 h and gradually decreased until 96 h (Figure S1A, Supporting Information). In contrast, the extract from the non‐treated Ti (control) showed no change in pH, maintaining values ≈7.6 throughout the observation period. The pH of the Mg–30Ca extract was significantly higher than that of the control at all time points, indicating that dissolution of the Mg–30Ca coating provided a sustained alkaline environment. In addition, no pH elevation was observed in the ion‐supplemented media compared with the control (Figure S1B).

### 3.2 Cytotoxicity Assessment of Mg^2+^ on MC3T3‐E1 Cell Proliferation

**Figure 2:**
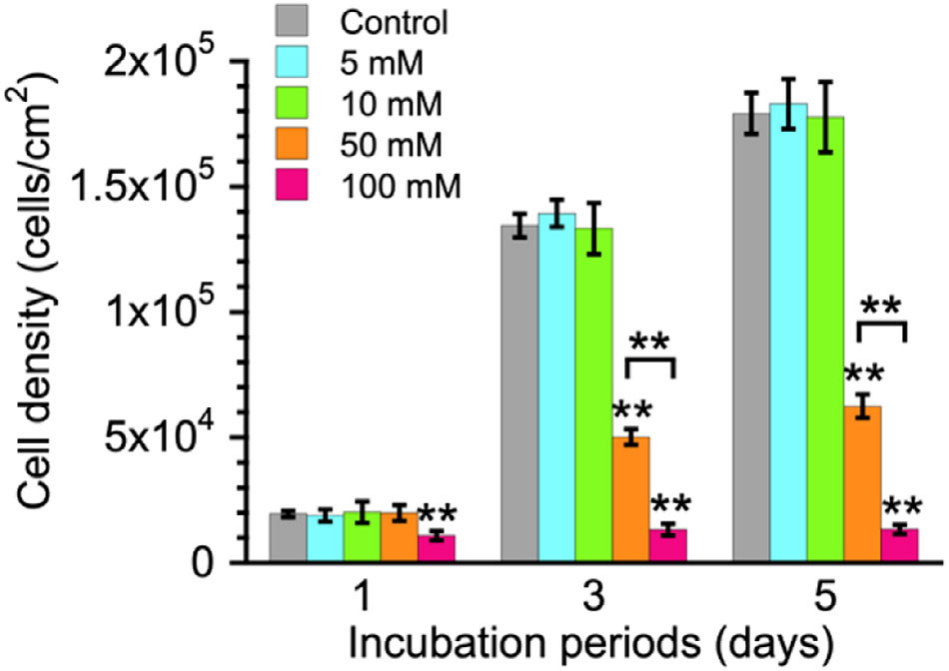
Cell density of MC3T3‐E1 cells after 1, 3, and 5 days of incubation in cell culture media containing 0.9 (control), 5, 10, 50, and 100 mm Mg^2+^. Data presented as mean ± SD, n = 5, *P*‐values are calculated using one‐way ANOVA followed by Tukey's post hoc test, * represents *p *< 0.05, ** represents *p *< 0.01 compared to the control group at each incubation period, horizontal bars indicate significant differences among other groups.

**Figure** **2** shows the results of the cellular proliferation assay for MC3T3‐E1 cells cultured in media containing 0.9–100 mm Mg^2+^. Since the Mg^2+^ concentration in the original medium is ≈0.9 mm, a 0 mm Mg^2+^ group was not included in this study. There was no significant difference in cell density between the control and 10 mm Mg^2+^. In contrast, 50 mm Mg^2+^ resulted in a significantly lower cell density, and 100 mm further reduced cell density after 3 and 5 days of incubation. Based on the method reported by Wang et al. and ISO 10993–5, we adopted the criterion that cell viability greater than 75% can be considered non‐cytotoxic for medical devices.^[^ ^22^, ^23^ ^]^ Concentrations over 50 mm Mg^2+^ resulted in less than 75% cell viability compared to 0.9 mm Mg^2+^ at days 3 and 5 (Figure S2, Supporting Information).

### 3.3 Viability Assessment of MC3T3‐E1 Cells in Response to Mg^2+^, Ca^2+^, and pH

**Figure 3:**
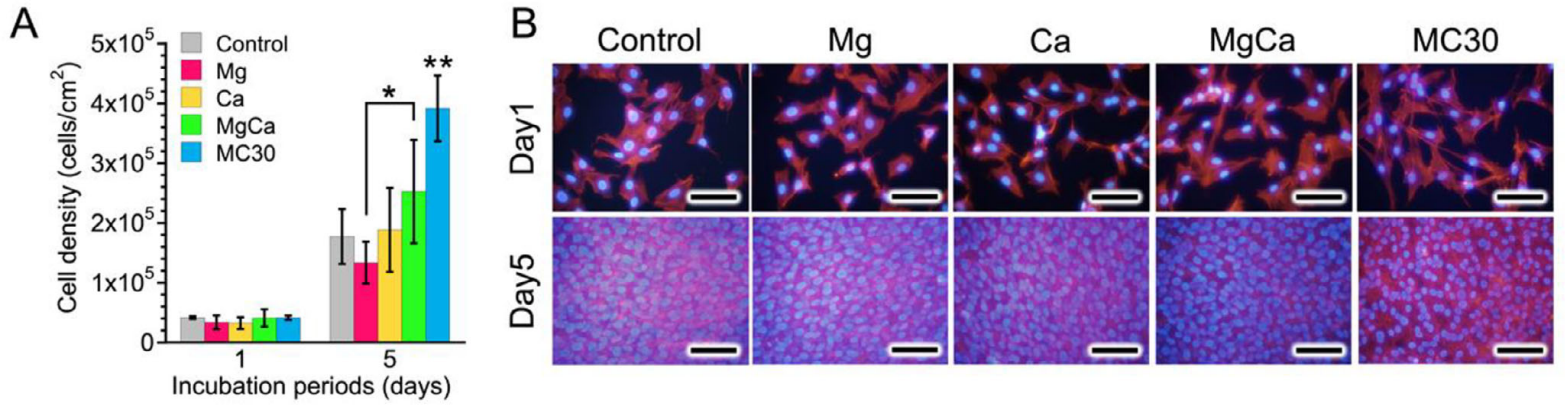
Cell density of MC3T3‐E1 cells after 1 and 5 days of incubation in each group A). Fluorescence images of the MC3T3‐E1 cells after incubation for 1 and 5 days B). F‐actin (red) and nuclei (blue) of attached cells were stained with phalloidin and Hoechst (scale bars = 100 µm). Data presented as mean ± SD, n = 5, *P*‐values are calculated using one‐way ANOVA followed by Tukey's post hoc test, * represents *p *< 0.05, ** represents *p *< 0.01 compared to the control group at each incubation period, horizontal bars indicate significant differences among other groups.

Cell viability test was conducted using cell culture media with Mg^2+^ and Ca^2+^ concentrations and pH values as shown in Table 1. The densities of the MC3T3‐E1 cells incubated in each cell culture medium increased with the incubation time (**Figure** **3**A). After 1 day of incubation, no significant differences in cell density were observed among the samples. After 5 days of incubation, no significant difference in cell density was observed between the Mg group and the control group. Similarly, when comparing the control and Ca groups, cell densities were nearly identical. No significant difference in cell density was observed among the MgCa, control, and Ca groups. In contrast, the MgCa group showed significantly higher cell density compared to the Mg group. Furthermore, the cell density of MC3T3‐E1 cells in the MC30 group was significantly higher compared to that in the other groups after 5 days of incubation. Fluorescent images (Figure 3B) obtained after 1 day of incubation show that cells were well spread in all groups. Moreover, the cells exhibited excellent adhesion, spreading, and proliferation to the extent that individual cells could no longer be clearly distinguished after 5 days of incubation.

To clarify the independent effect of pH elevation on cell proliferation, MC3T3‐E1 cells were cultured in Mg, Ca, and MgCa groups (Table 1), and in media further adjusted to pH 9.5. As shown in Figure S3A–C (Supporting Information), cell proliferation increased with incubation time in all groups, but the extent of proliferation differed among conditions. At pH 8, no significant difference in cell density was observed between the control and only ion‐supplemented groups after either 1 or 5 days. However, when the pH of the ion‐supplemented media was further increased from 8 to 9.5, a significant increase in cell density was observed in all‐ion‐supplemented groups (Mg, Ca, and MgCa) compared with control groups.

### 3.4 Alkaline Phosphatase Activity as a Marker of Osteogenic Response to Mg^2+^, Ca^2+^, and pH

**Figure 4:**
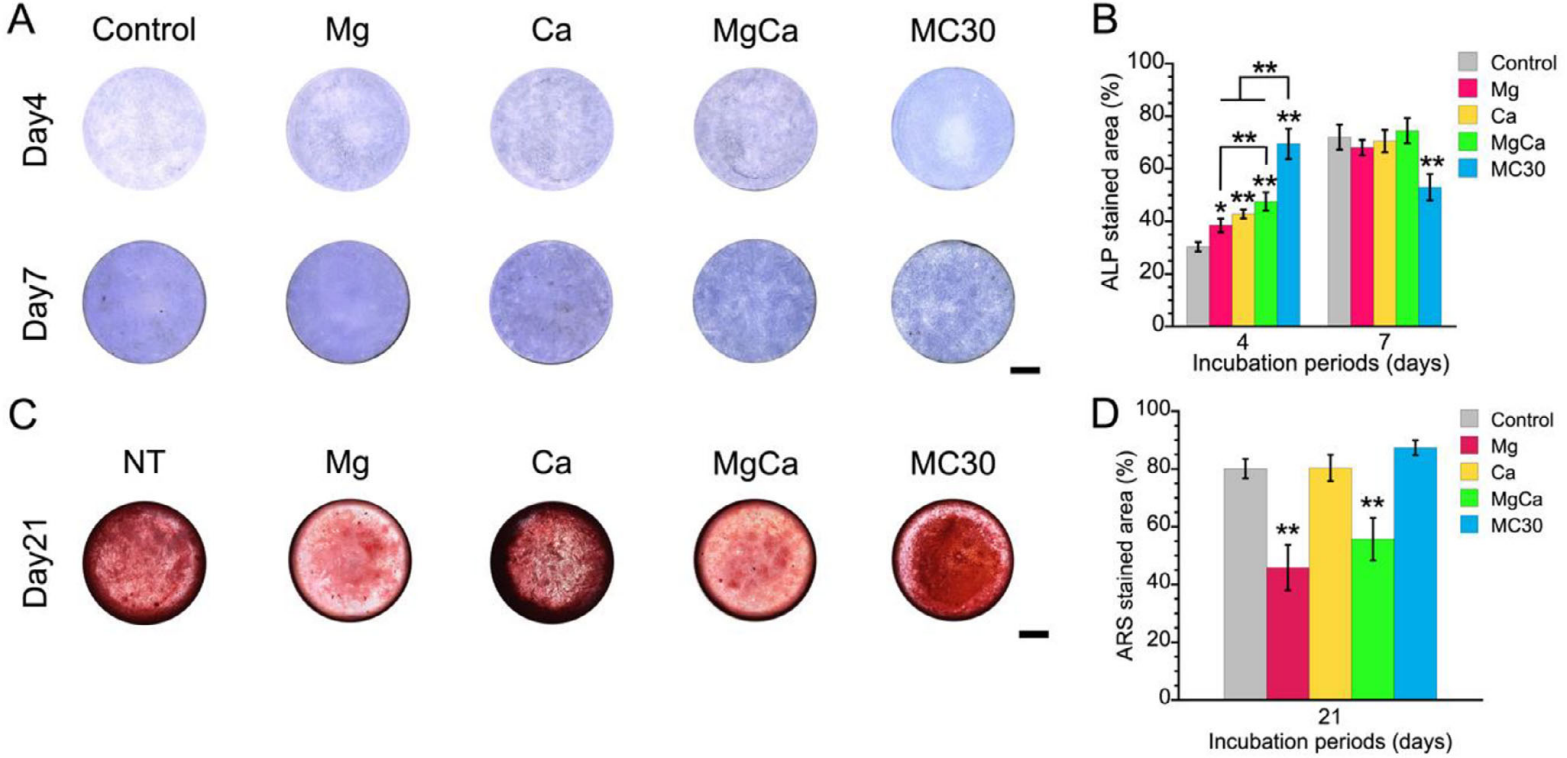
Optical microscope images of ALP‐stained area (blue) of each group after 4 and 7 days of incubation A) and the percentage of ALP‐stained area B). ARS‐stained images of the calcified deposition area (red) of each group after 21 days of incubation C). ARS‐stained area for quantification of mineral nodule formation D). (scale bar = 1 mm) Data presented as mean ± SD, n = 3–4, *P*‐values are calculated using one‐way ANOVA followed by Tukey's post hoc test, * represents *p *< 0.05, ** represents *p *< 0.01 compared to the control group at each incubation period, horizontal bars indicate significant differences among other groups.

ALP‐stained areas of MC3T3‐E1 cells of each group are shown in **Figure** **4**A, and the corresponding percentages of ALP‐positive areas are presented in Figure 4B. After 4 days of incubation, the Mg group exhibited a significantly larger ALP‐stained area compared to the control group, while no significant difference was observed between the two groups at 7 days. The Ca group also showed a significantly higher percentage of ALP‐stained area than the control group at 4 days, whereas their levels were comparable at 7 days. Furthermore, the MgCa group exhibited a significantly greater ALP‐stained area than both the control and Mg groups at 4 days. At day 7, the ALP‐stained area of the MgCa group was comparable to those of the control, Mg, and Ca groups. The MC30 group exhibited a significantly larger ALP‐stained area than the other groups at day 4, but a smaller area at day 7. To evaluate whether these relatively early time points were appropriate, additional ALP staining was performed for the control group at culture days 5, 10, and 15. As shown in Figure S4 (Supporting Information), ALP activity reached its maximum level on day 10 and significantly decreased on day 15 compared with days 5 and 10. In addition, to clarify the independent effect of pH elevation on ALP activity, MC3T3‐E1 cells were cultured in differentiation‐inducing media and in differentiation‐inducing media further adjusted to pH 8.7. As shown in Figure S5 (Supporting Information), the control (pH 8) exhibited a larger ALP‐stained area than the pH‐adjusted one. Furthermore, Figure S6 (Supporting Information) shows ALP‐stained areas of MC3T3‐E1 cells of MC30 group without pH adjustment (initial pH 9.5). Very few ALP‐stained areas were observed after 4 and 7 days of incubation.

### 3.5 Evaluation of Osteogenic Mineralization via Alizarin Red S Staining in Response to Mg^2+^, Ca^2+^, and pH

ARS‐stained areas of MC3T3‐E1 cells incubated in each medium (Table 2) are shown in Figure 4C, and the quantitative analysis is presented in Figure 4D. After 21 days of incubation, the Mg group exhibited a significantly smaller ARS‐stained area compared to the control group. In contrast, the Ca group showed a mineralization level comparable to that of the control group. The MgCa group displayed a significantly lower level of mineralization than both the control and Ca groups. It exhibited a higher mineralization level than the Mg group, the difference was not statistically significant. Furthermore, the MC30 group demonstrated a high level of mineralization comparable to the control and Ca groups, despite having similar Mg^2+^ and Ca^2+^ concentrations to the Mg and MgCa groups. No ARS‐stained area was observed in the pH‐adjusted control group (pH 9.3), whereas the surface was fully covered with ARS staining in the pH‐unadjusted control group (Figure S7, Supporting Information).

## 4 Discussion

In this study, we investigated the effects of Mg^2+^, Ca^2+^, and pH elevation resulted from the degradation of Mg‐30Ca coating as extracellular factors regulating the viability and osteogenic differentiation of MC3T3‐E1 cells in vitro. To evaluate the specific effects of ions released from metal, we compared these results with those obtained using media supplemented with corresponding concentrations of Mg and Ca salts. It is reported that MC3T3‐E1 cells are a well‐established model for studying osteoblast differentiation, as they are easy to culture, exhibit robust proliferation, and possess strong osteogenic potential under appropriate conditions.^[^ ^24^ ^]^

Cell proliferation of MC3T3‐E1 cells was not significantly affected at Mg^2^⁺ concentrations between 0.9 and 10 mm but was inhibited at 50 and 100 mm (Figure 2). The MC30 group showed significantly higher cell density compared to other groups after 5 days of incubation (Figure 3). Early ALP activity was enhanced in the MC30 group, while mineralization was comparable to that in the control and Ca groups (Figure 4). Figure S4B (Supporting Information) shows that in the control group, the ALP‐stained area increased from day 5 to day 10 and then significantly decreased by day 15. This pattern indicates that ALP activity in MC3T3‐E1 cells peaks around day 10 and subsequently declines, likely as the culture approaches confluence. Therefore, it was reasonable to evaluate ALP activity at the relatively early time points (days 4 and 7) to detect the earlier ALP peak accelerated by MC30. These results indicate that the MC30 group achieved superior osteogenic responses compared to the other groups. In general, Mg^2+^ plays an important role in regulating osteoblast function.^[^ ^25^ ^]^ Previous studies have demonstrated that Mg^2+^ exerts concentration‐dependent effects on osteoblast function, with 10 mm defined as the critical dose that does not adversely affect cell growth.^[^ ^26^ ^]^ Our cytotoxicity test results suggest that Mg^2+^ concentrations between 0.9–10 mm are favorable for cell proliferation (Figure 2). Accordingly, our results do not contradict previous findings, and a concentration of 4–5 mm Mg^2+^ was adopted for subsequent experiments. In our cell proliferation experiment, 5 mm Mg^2+^ alone did not significantly affect the proliferation of MC3T3‐E1 cells. In terms of osteogenesis, Mg^2+^ promotes the differentiation of mesenchymal stem cells into osteoblasts and enhances the expression of osteogenic markers, including OCN, OPN, ALP, RUNX2, IGF‐1, PGC‐1α, and COL10A1.^[^ ^27^ ^]^ ALP is an early marker of osteoblast activity, with its expression increasing during the initial stages of mineralization. ALP‐staining is a widely used method for evaluating early osteogenic differentiation of mesenchymal stem cells and pre‐osteoblasts. In this assay, ALP activity is visualized through the deposition of a coloured precipitate at enzyme‐active sites, enabling qualitative and semi‐quantitative assessment of osteogenic potential. Previous studies have reported that early osteogenic markers, such as ALP activity and osteocalcin expression, are significantly upregulated at Mg^2+^ concentrations of 0.5–2.0 mm, but significantly downregulated at concentrations of 8 mm and above after 10 days of incubation.^[^ ^28^, ^29^ ^]^ Based on our results, ≈4 mm Mg^2+^ enhanced ALP activity compared to control groups containing 0.9 mm Mg^2+^ at the early stage of osteogenic differentiation (Figure 4). Furthermore, Mg^2+^ supports mineralization by increasing amorphous calcium phosphate (ACP) deposition. It is reported that mineralization activity was increased at 1–2 mm, while higher concentrations (≥ 4 mm) led to a decline.^[^ ^28^ ^]^ These findings indicate that Mg^2^⁺ promotes osteoblast proliferation and early differentiation at low to moderate concentrations (1–2 mm). In contrast, excessive Mg^2+^ (≥ 4 mm) inhibits mineralization, likely due to interference with calcium phosphate crystal formation or altered cellular signaling, suppressing osteogenic signaling pathways, and disrupting cellular ionic homeostasis, ultimately impairing osteoblast differentiation and mineralization.^[^ ^30^, ^31^, ^32^ ^]^ In agreement with these reports, our ARS staining analysis indicated that a 4 mm Mg^2+^ concentration inhibited mineralization. Thus, Mg^2+^ exhibits a concentration‐dependent dual effect on osteoblasts, enhancing early differentiation at optimal levels while suppressing matrix mineralization at elevated concentrations. According to previous reports, Mg^2+^ concentrations of 1–2 mm are suitable for promoting ALP activity and mineralization, whereas concentrations above 8 mm inhibit ALP activity, and concentrations above 4 mm suppress mineralization.^[^ ^28^, ^29^ ^]^ In this study, increasing the Mg^2+^ concentration from 0.9 to 4–5 mm did not affect cell proliferation, but 4–5 mm Mg^2+^ suppressed mineralization while promoting ALP activity at the early stage of differentiation (after 4 days of incubation). We believe that 4–5 mm Mg^2+^ suppressed mineralization by interference with calcium phosphate crystal formation. Excess Mg^2+^ may also disturb cellular ionic balance, leading to impaired late‐stage osteoblast differentiation.

Extracellular Ca^2+^ has been shown to control a variety of functions, including cell proliferation, differentiation, and mineralization.^[^ ^33^ ^]^ In addition, extracellular Ca^2+^ regulates osteoblastic proliferation and differentiation by changing the expression of specific Ca^2+^‐channel isoforms on osteoblasts and has been accepted as a coupling factor between osteoclasts and osteoblasts.^[^ ^34^ ^]^ Previous studies have reported that Ca^2+^ concentrations of 2–6 mm promote osteoblast proliferation, while concentrations in the range of 0–4 mm enhance ALP activity in a concentration‐dependent manner.^[^ ^35^ ^]^ Additionally, Ca^2+^‐dependent mineralization has been observed in the concentration range of 1–10 mm. High extracellular Ca^2+^ concentrations (> 10 mm) may suppress ALP activity by promoting premature matrix mineralization, accelerating osteoblast differentiation stages, or inducing cellular stress responses that interfere with normal differentiation pathways. Thus, the optimal concentration range varies depending on the type of evaluation, and taken together, a Ca^2+^ concentration in the range of 2–4 mm appears to be optimal. In this study, a slight increase in Ca^2+^ concentration from 2.2 to 3 mm had no effect on cell proliferation, while an increase from 1.3 to 2.3 mm did not influence mineralization but significantly increased ALP‐stained area at day 4. Even a slight increase in Ca^2+^ concentration was sufficient to influence osteogenic differentiation.

The combined effects of Mg^2+^ and Ca^2+^ ions on bone regeneration remain poorly understood, and only limited studies have been reported to date. One previous study demonstrated that the co‐presence of Mg^2+^ with Ca^2+^ reduced the adsorption of key inflammatory proteins such as C‐reactive protein and bleomycin hydrolase compared to Ca^2+^ alone, suggesting that Mg^2+^ may mitigate Ca^2+^‐induced inflammatory responses by using Ca and Mg in sol–gel coatings doped with mixtures of CaCl_2_ (0.5%) and MgCl_2_ (0.5, 1, and 1.5%).^[^ ^36^ ^]^ In the present study, the combined supplementation of Ca^2+^ and Mg^2+^ resulted in enhanced cell proliferation compared to Mg^2+^ alone. Although the addition of Ca^2+^ partially restored mineralization suppressed by high concentrations of Mg^2+^, the level of mineralization remained significantly lower than that of the control group. The inhibitory role of Mg^2+^ in mineralization through stabilization of ACP and suppression of its transformation to apatite has been well reported in previous studies. Furthermore, it is reported that Mg^2+^ and Ca^2+^ competitively adsorb onto apatite crystal surfaces, where Mg^2+^ preferentially binds to growth sites and inhibits crystal growth by stabilizing ACP. However, Ca^2+^ exhibits a higher affinity for these sites and can replace the adsorbed Mg^2+^, restoring apatite formation.^[^ ^37^ ^]^ These findings suggest that the balance between Mg^2+^ and Ca^2+^ concentrations regulates the nucleation and growth of calcium phosphate minerals during mineralization. Therefore, in this study, the coexistence of Ca^2+^ and Mg^2+^ is considered to have alleviated the inhibitory effect of Mg^2+^ on mineralization by establishing a favorable ionic balance for apatite formation. Notably, ALP activity at day 4 in the MgCa group was higher than that in both the control and Mg groups, indicating a promotive effect on early‐stage osteogenic differentiation. These findings suggest that, in addition to the individual roles of Mg^2+^ and Ca^2+^ in osteogenesis, their coexistence may exert synergistic effects on specific stages of the bone formation process.

Extracellular pH is also a critical factor in osteogenesis. A previous study reported that osteoblast‐like cells showed maximal proliferation at pH 8.0–8.4, and that mineralization was greater at pH 7.8 compared to pH 7.4.^[^ ^38^ ^]^ The enhancement of osteoblast‐like cell proliferation and differentiation under alkaline conditions was attributed to increased mitochondrial activity, elevated ATP synthesis, activation of the p38 MAPK pathway, and the physiologically favorable role of alkaline pH in bone formation processes.^[^ ^39^ ^]^ In this study, the effects of Mg^2+^, Ca^2+^, and extracellular pH on osteogenic activity were evaluated separately in terms of cell proliferation, ALP activity, and mineralization. The MC30 group exhibited significantly enhanced cell proliferation compared to the other groups, despite having similar Mg^2+^ and Ca^2+^ concentrations as the MgCa group. This suggests that the elevated pH in MC30, which was ≈1.5 units higher, contributed to the increased proliferation. The high pH observed in the MC30 medium can be explained by the following reaction equation. Mg^2+^ and Ca^2+^ of MC30 derived from the dissolution of metallic Mg and Ca. During dissolution, metallic Mg and Ca changed into their ions, and electrons were generated (Equations (1) and (2)). These electrons reacted with water in the original medium, resulting in hydrogen generation and an increase in pH (Equation (3)). Therefore, MC30 had a higher pH value compared to other groups.

(1) $$\mathrm{Mg}\to Mg^{2+}+2e^{-}$$

(2) $$\mathrm{Ca}\to Ca^{2+}+2e^{-}$$

(3) $$4H_{2}O+4e^{-}\to 2H_{2}+4OH^{-}$$

The initial pH of the MC30 medium was ≈9.5 (Table 1) due to the degradation of the Mg–30Ca coating. However, upon incubation in a CO_2_ incubator (5% CO_2_, 37 °C), the pH was expected to decrease. This decrease in pH can be attributed to the dissolution of CO_2_ into the medium, which leads to the formation of carbonic acid (H_2_CO_3_) and the subsequent release of protons (H^+^), thereby lowering the pH according to the following reaction (Equation (4)).

(4) $$CO_{2}+H_{2}O⇌H_{2}CO_{3}⇌H^{+}+HC{O_{3}}^{-}$$

Normally, a relatively higher pH value (pH 8.0‐8.4) promotes osteoblast proliferation.^[^ ^36^ ^]^ The initial pH of 9.5 for the MC30 group is considered too alkaline for cell proliferation; however, the pH is expected to decrease during incubation, shifting toward a more favorable range. Figure S1 (Supporting Information) indicates that pH elevation did not occur by ion supplementation alone but rather occurred specifically through the dissolution of the Mg–30Ca coating. Dissolution behavior of the Mg–30Ca coating in the incubator revealed that, within 1–2 days, the surrounding environment of the coating became moderately alkaline, which is favorable for cell proliferation. This finding suggests that the dissolution of the Mg–30Ca coating provides the biologically compatible alkaline microenvironment that supports cellular responses. As shown in Figure S3A–C (Supporting Information), ion supplementation alone did not significantly enhance cell proliferation compared with the control. However, when the pH of the ion‐supplemented media was adjusted to 9.5, a significant increase in cell density was observed in all ion‐supplemented groups. These results indicate that the enhanced cell proliferation observed in the Mg–30Ca extract (MC30) is primarily attributed to the elevated pH. Based on our results, enhancement of cell proliferation in the MC30 group was considered to result from not only the combined effects of elevated Mg^2+^ and Ca^2+^ concentrations but also increased pH.

In addition, ALP activity was markedly higher in the MC30 group compared to the other groups, including the MgCa group, having similar Mg^2+^ and Ca^2+^ concentration to MC30 group. Given that ALP is a marker of early‐stage osteogenic differentiation, this result suggests that a moderately alkaline environment can enhance early osteogenic differentiation. Furthermore, the ALP activity in the Mg, Ca, and MgCa groups was higher than that in the control group. Therefore, the highest ALP activity in MC30 groups was caused by the combined effects of elevated Mg^2+^ and Ca^2+^ concentrations along with increased pH. In general, moderately alkaline conditions are considered to contribute to osteoblast differentiation through enhanced expression of the ALP gene and by providing a more favorable environment for calcium phosphate formation.^[^ ^40^ ^]^ In contrast, the medium with only an increased pH had lower ALP activity compared to the control group (Figure S5B, Supporting Information). In this study, ALP activity was measured by ALP staining based on the BCIP/NBT method, which involves enzymatic dephosphorylation of BCIP by ALP during the staining process.^[^ ^41^ ^]^ Higher pH conditions would promote inorganic phosphate (Pi) accumulation, which can competitively inhibit ALP and interfere with ALP staining.^[^ ^42^, ^43^, ^44^ ^]^ As evidence of this, Figure S6 (Supporting Information) demonstrates that ALP staining was not effective under unadjusted high pH conditions, indicating that high alkalinity may interfere with the visualization of enzymatic activity. Therefore, it is considered that the lower ALP activity in the medium with only an increased pH was caused by the increasing Pi. In this study, the pH value of the MC30 group was adjusted from 9.3 to 8.7 during the evaluation for ALP activity to minimize the pH‐induced inhibitory effect of accumulated Pi on ALP activity. Furthermore, Figure 4A,B indicate that the increasing Pi by pH elevation in the medium reacted to counter cations such as Mg^2+^ and Ca^2+^ presented in the MC30 group for further reducing the inhibitory effect of accumulated Pi.

Mineralization in the MC30 group was significantly improved compared to the MgCa group, despite having similar Mg^2+^ and Ca^2+^ concentrations (Figure 4C,D). On the other hand, adjustment of the medium pH alone did not induce mineralization to the same extent as the control (Figure S7, Supporting Information). These results suggest that both ionic effects (Mg^2+^ and Ca^2+^ release) and pH elevation are required for promoting mineralization, as comparable mineralization was observed only in the MC30 group, where both factors coexist. As previously discussed, the pH of the MC30 medium was expected to decrease during incubation; however, the resulting pH remained within a range considered favorable for the differentiation of MC3T3‐E1 cells. While high Mg^2+^ levels are generally associated with suppressed mineralization, the elevated pH in the MC30 group appears to have counteracted this inhibitory effect, leading to mineralization levels comparable to those observed in the untreated (control) group. It has been reported that mineralization downregulates ALP activity, as the onset and extent of ALP decline closely mirror the timing and extent of calcium accumulation during osteogenic differentiation.^[^ ^24^, ^45^ ^]^ Based on the results of this study, it is suggested that a relatively higher pH may shift the peak of ALP activity to an earlier stage, thereby promoting subsequent mineralization.

This study has several limitations. First, biodegradable metallic materials such as the Mg–30Ca coating release hydrogen gas during in vivo degradation.^[^ ^46^ ^]^ The effects of hydrogen generation were not investigated in this study. Second, although the initial pH of the culture media was adjusted, real‐time monitoring of pH fluctuations during incubation was not performed. Since pH can dynamically change during cell culture, continuous monitoring would be valuable to precisely correlate pH variations with cellular responses. Third, while the effects of combined Mg^2+^ and Ca^2+^ supplementation were evaluated, systematic optimization of their concentration ratios was not conducted. A more detailed analysis of different Mg^2+^:Ca^2+^ ratios could provide deeper insights into the optimal ionic environment for osteogenesis. Finally, the lack of quantitative analysis of ALP and ARS staining has been recognized as a limitation of the present study. In future work, quantitative evaluations of staining intensity and calcium deposition will be included to enable more precise assessment of osteogenic differentiation and to further validate the findings of this study.

In conclusion, Mg^2+^ and Ca^2+^ released from metallic Mg‐30Ca coating, as compared to those derived from soluble salts, did not exhibit an adverse effect on cell behaviors. Rather, the elevation of pH caused by ion dissolution played an important role in the differentiation of osteoblast‐like cells.

## 5 Conclusion

In this study, we simulated the local environment after degradation of a Mg–Ca‐based coating by using extraction solutions containing dissolved ions and elevated pH. Our findings demonstrated that moderate Mg^2+^ concentrations (≈4–5 mm) did not adversely affect osteoblast proliferation and even enhanced ALP activity during early differentiation. Although these Mg^2+^ levels suppressed mineralization, the addition of Ca^2+^ led to a slight improvement. Similarly, 2.3–3 mm Ca^2+^ also did not adversely affect osteoblast proliferation and even enhanced ALP activity during early differentiation. The addition of Ca^2+^ to Mg^2+^ enhanced cell proliferation and ALP activity at the early stage of differentiation compared to Mg^2+^ alone. Furthermore, in the MC30 group, which exhibited a relatively high pH, both osteoblast proliferation and ALP activity were enhanced, and mineralization was substantially restored despite having the same Mg^2+^ concentration as the Mg and MgCa groups. These results suggest that not only ion composition but also the resulting pH environment plays a critical role in osteogenic responses. Overall, the Mg–Ca coating showed promising potential as a bioactive, degradable surface for dental implants, provided that the dissolution behavior and local pH changes are appropriately controlled.

## Conflict of Interest

The authors declare no conflict of interest.

## Supporting information



Supporting Information

## Data Availability

The data that support the findings of this study are available from the corresponding author upon reasonable request.
