# Supplementary material for: Osteogenic Effects of Ion Released from Biodegradable Metallic Magnesium and Calcium Coating
Source: Adv Biol (Weinh). 2025 Nov 12;9(12):e00401. doi: 10.1002/adbi.202500401 (PMC12712752; doi:10.1002/adbi.202500401)
Supplement: Supplementary file 1 — Supporting Information [file ADBI-9-e00401-s001.docx]

Supporting Information

**Osteogenic Effects of Ion Released from Biodegradable Metallic Magnesium and Calcium Coating**

Risa Miyake*, Masaya Shimabukuro*, Masahiko Terauchi, Eriko Marukawa, and Masakazu Kawashita

**Table S1.** Actual Mg^2+^ and Ca^2+^ concentrations of the cell culture medium and the extract from the Mg–30Ca-coated Ti measured after immersion in the medium.

| Medium | Mg^2+^ concentration (mM) | Ca^2+^ concentration (mM) |
| --- | --- | --- |
| Cell culture medium | 0.91 | 2.27 |
| Extraction of Mg–30Ca coating | 5.31 | 2.94 |

**Table S2.** Actual Mg^2+^ and Ca^2+^ concentrations of the differentiation-inducing medium and the extract from the Mg–30Ca-coated Ti measured after immersion in the differentiation-inducing medium.

| Medium | Mg^2+^ concentration (mM) | Ca^2+^ concentration (mM) |
| --- | --- | --- |
| Differentiation-inducing medium | 0.89 | 1.26 |
| Extraction of Mg–30Ca coating | 4.02 | 2.33 |


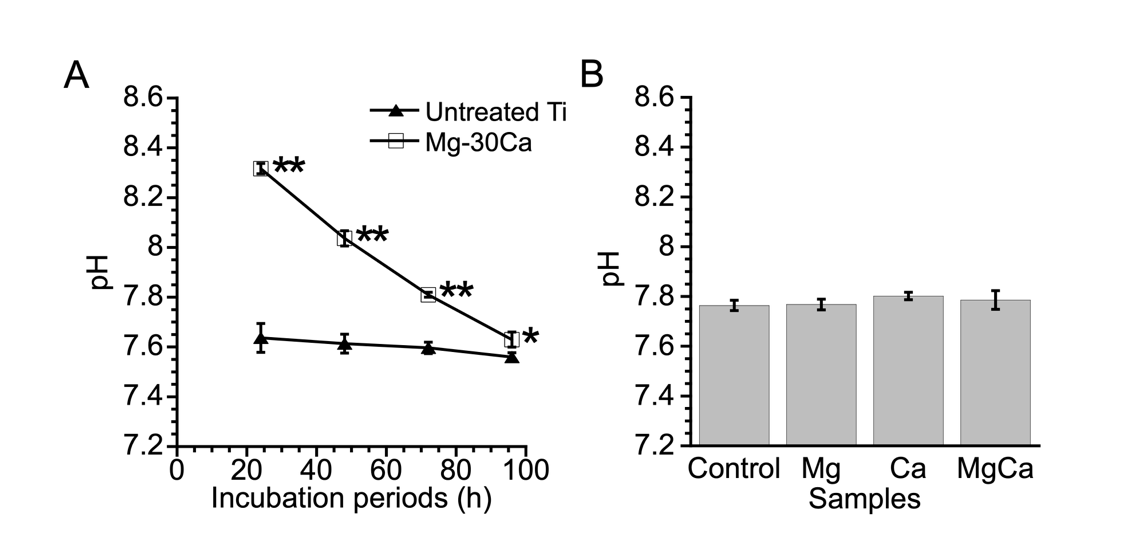


**Figure S1.** Temporal changes in the pH of the extraction during immersion of Mg–30Ca coated and untreated Ti disks. Each disk was immersed in 1 mL of the medium under 5% CO_2_ at 37 ℃. The medium was replaced every 24 h, and the pH of each extract was measured at 24, 48, 72, and 96 h to monitor changes associated with coating dissolution (A). pH of the ion-supplemented medium after 24 h incubation under 5% CO_2_ at 37 ℃. (B). Data presented as mean ± SD, n=3, *P*-values are calculated using one-way ANOVA followed by Tukey’s post hoc test, * represents *p*<0.05, ** represents *p*<0.01 compared to the control group at each incubation period.


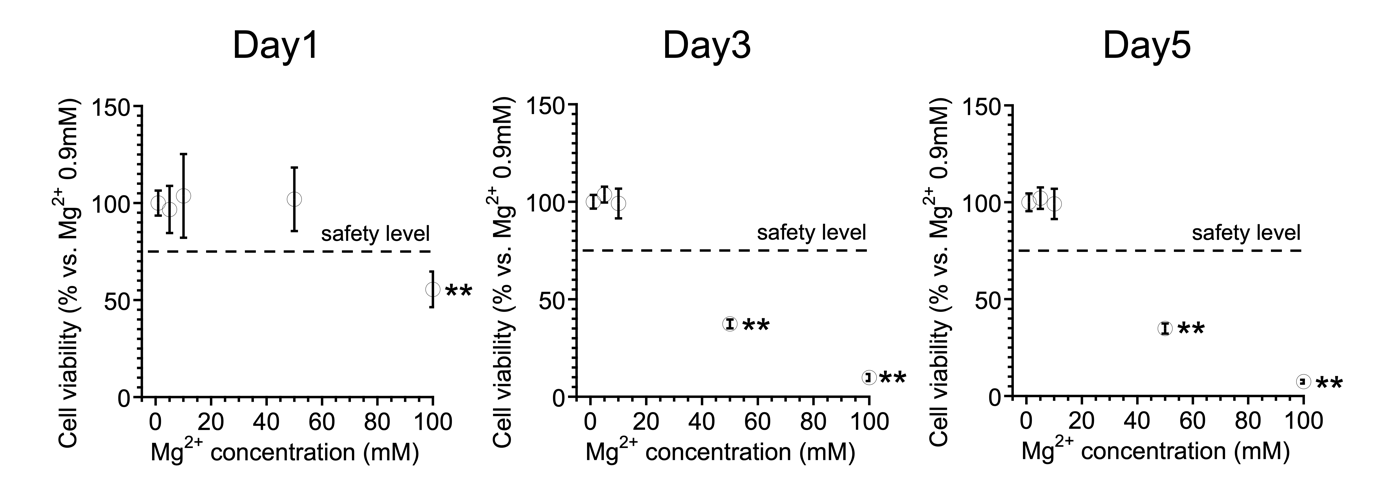


**Figure S2.** Cell viability of MC3T3-E1 cells after 1, 3, and 5 days of incubation in cell culture media containing 0.9, 5, 10, 50, and 100 mM Mg^2+^. Data presented as mean ± SD, n=3, *P*-values are calculated using one-way ANOVA followed by Tukey’s post hoc test, * represents *p*<0.05, ** represents *p*<0.01 compared to the control group at each incubation period.


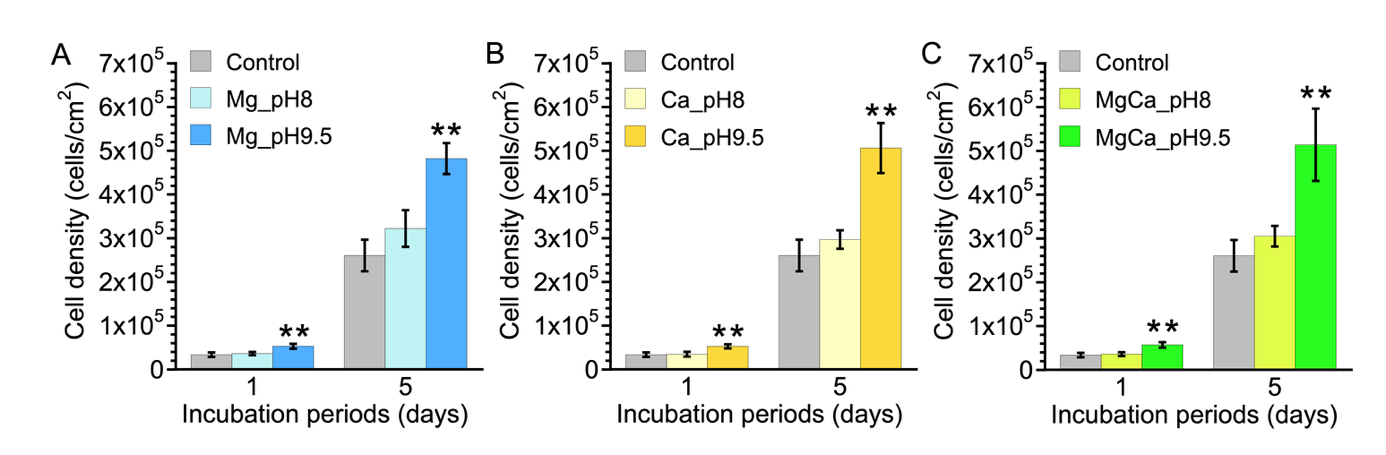


**Figure S3.** Cell proliferation of MC3T3-E1 cells cultured in the cell culture medium (control, pH 8), ion-supplemented (Mg, Ca, and MgCa), and pH-adjusted media. Cell density under each ion-supplemented condition (Mg, Ca, and MgCa) at pH 8 and pH 9.5 after 1 and 5 days of incubation. (A-C). Data presented as mean ± SD, n=5, *P*-values are calculated using one-way ANOVA followed by Tukey’s post hoc test, * represents *p*<0.05, ** represents *p*<0.01 compared to the control group at each incubation period.


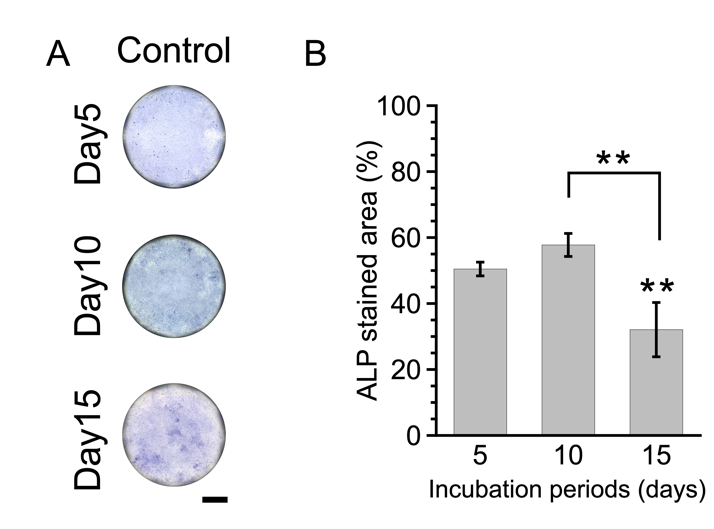


**Figure S4.** Optical microscope images of ALP-stained area (blue) of control group after 5, 10, and 15 days of incubation (A) and the percentage of ALP-stained area (B). Data presented as mean ± SD, n=3, *P*-values are calculated using one-way ANOVA followed by Tukey’s post hoc test, * represents *p*<0.05, ** represents *p*<0.01 compared to the Day 5 group at each incubation period, horizontal bars indicate significant differences among other groups.


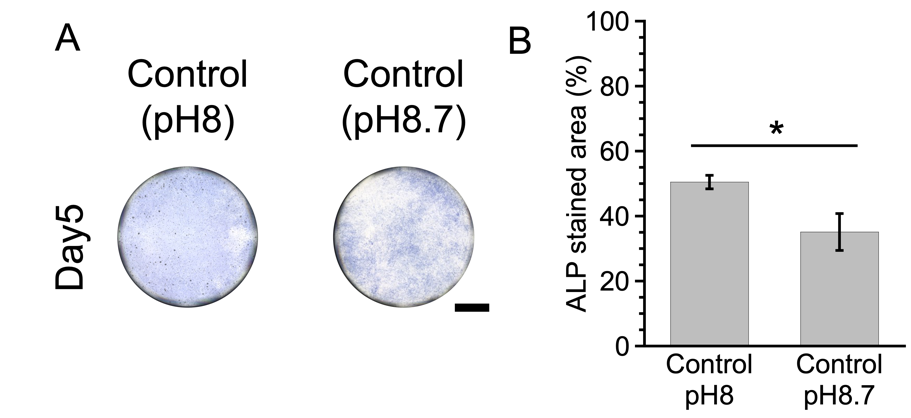


**Figure S5.** Optical microscope images of ALP-stained area (blue) of each group after 5 days of incubation (A) and the percentage of ALP-stained area (B). Data presented as mean ± SD, n=3, *P-*values are calculated using one-way ANOVA followed by Tukey’s post hoc test, * represents *p*<0.05 among the groups.


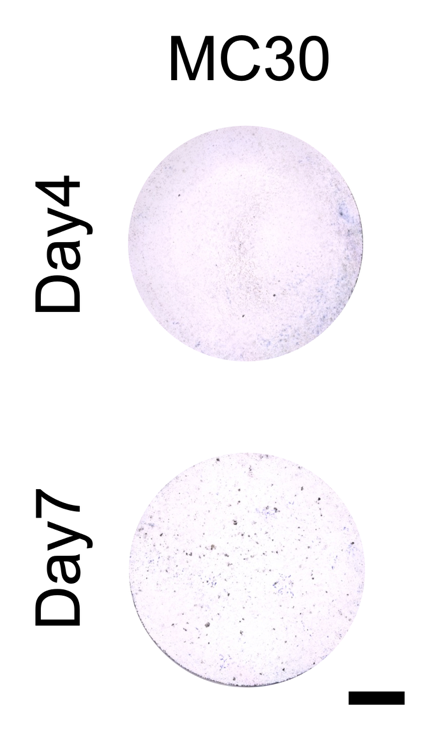


**Figure S6.** Optical microscope images of ALP-stained area (blue) of MC30 (pH 9.3) after 4 and 7 days of incubation. (scale bar = 1 mm).


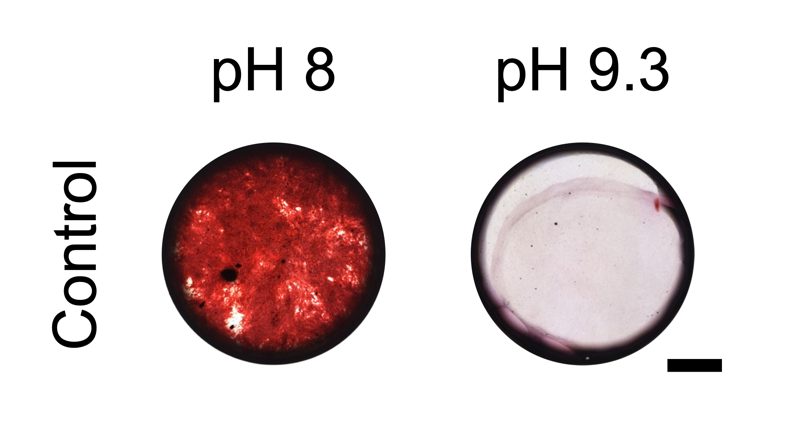


**Figure S7.** ARS-stained images of the calcified deposition area (red) of control cells under pH 8.0 and pH 9.3 after 21 days of incubation (scale bar = 1 mm).
